# *Prnp* Deletion Mitigates Muscle Fiber Type‐Specific Sarcopenia Induced by Prion Infection in Mice

**DOI:** 10.1002/iid3.70425

**Published:** 2026-04-13

**Authors:** Wenduo Liu, Yong‐Chan Kim, Sae‐Young Won, Thi Thu Trang Kieu, Sung Ho Kook, Byung‐Hoon Jeong, Sang Hyun Kim

**Affiliations:** ^1^ College of Physical Education Beihua University Jilin City Jilin China; ^2^ Department of Sports Science, College of Natural Science Jeonbuk National University Jeonju Republic of Korea; ^3^ School of Life Sciences and Biotechnology Gyeongkuk National University Andong Republic of Korea; ^4^ Department of Bioactive Material Sciences, Research Center of Bioactive Materials Jeonbuk National University Jeonju Republic of Korea; ^5^ Korea Zoonosis Research Institute Jeonbuk National University Iksan Republic of Korea

**Keywords:** mitochondria, *Prnp* knockout, PrP^C^ protein, sarcopenia, scrapie strain ME7, skeletal muscle

## Abstract

Recent studies have shown that significant expression of PrP^C^ protein is also present in skeletal muscle, and it plays a significant role in maintaining skeletal muscle homeostasis. Although the expression of PrP^C^ in skeletal muscle has been clarified, the effects of PrP^Sc^‐mediated prion protein infection on sarcopenia in mice and its potential regulatory mechanisms remain unclear. This study investigated the role of PrP^C^ in Prion‐induced sarcopenia, using an animal model of prion disease based on intraperitoneal injection of the scrapie strain ME7 into wild‐type mice and Prnp knockout mice. The results indicate that prion infection‐induced sarcopenia exhibits muscle fiber type specificity, and that the lack of PrP^C^ can prevent prion protein infection‐induced sarcopenia, although the lack of PrP^C^ may lead to reduced mitochondrial‐endoplasmic reticulum homeostasis. These data provide novel evidence that prion infection affects skeletal muscle system health through myofiber‐specific mechanisms.

## 1 Introduction

Prion diseases are a group of fatal neurodegenerative disorders with a wide host range, including Creutzfeldt‐Jakob disease in humans, scrapie in sheep and goats, and bovine spongiform encephalopathy in cattle [1, 2, 3]. The pathological hallmark of prion diseases is the accumulation of abnormally folded prion proteins (referred to as PrP^Sc^), which arise from structural and conformational changes in endogenous normal prion proteins (referred to as PrP^C^) [4, 5]. For prion diseases, PrP^C^ is a prerequisite for PrP^Sc^ to exert its function. PrP^C^‐deficient animals are unable to transmit PrP^Sc^ [6].

PrP^C^ is strongly expressed in tissues of the central nervous system [7], but recent studies have shown that significant PrP^C^ expression also exists in skeletal muscle [8], and PrP^C^ protein expression levels exhibit specific differences across distinct muscle fiber types [PrP content differs among mouse muscles: gastrocnemius (Gas) > extensor digitorum longus (EDL) > tibialis anterior (TA) and soleus (Sol)] [9]. Furthermore, PrP^C^ exhibits significant effects in maintaining mitochondrial–endoplasmic reticulum (ER) homeostasis in skeletal muscle, inhibiting satellite cell senescence, and regulating oxidative stress [8]. Although the expression and partial functions of PrP^C^ in skeletal muscle have been partially elucidated, the effects of PrP^Sc^‐mediated prion infection on sarcopenia and the underlying regulatory mechanisms remain unclear.

Here, we investigated the role of PrP^C^ in prion‐induced sarcopenia using an animal model of prion disease based on intraperitoneal (i.p.) injection of the scrapie strain ME7 into wild‐type mice and *Prnp* knockout mice. We provide novel evidence that prion‐induced sarcopenia exhibits muscle fiber type specificity, and that the lack of PrP^C^ can prevent prion‐induced sarcopenia, although it may lead to reduced mitochondrial–ER homeostasis.

## 2 Materials and Methods

### 2.1 Animals

*Prnp* (encoding PrP^C^) knockout mice on an FVB background were purchased from The Jackson Laboratory (Bar Harbor, ME, USA) as detailed in previous publication [5]. Mice were randomly assigned to each experimental group. Mice were housed (fewer than five per cage) in air‐conditioned rooms (temperature: 18°C–22°C; humidity: 40%–60%) with a light/dark cycle (12 h:12 h) and food and water available ad libitum. C57BL/6J mice (6 weeks old) were obtained from Nara Biotech (Pyeongtaek, Gyeonggi, Korea).

### 2.2 Ethical Statements

All experimental procedures were approved by the Institute of Animal Care and Use Committee of Jeonbuk National University (JBNU 2020‐080). All efforts were made to minimize the number of animals used and their suffering.

### 2.3 Statistical Analysis

All data are presented as the mean ± standard deviation (SD) and were analyzed using GraphPad Software (Prism 10, MA, USA). Differences between two groups were analyzed by unpaired Student's *t*‐test. One‐way ANOVA followed by the Tukey post hoc test was used for multiple comparisons among more than two groups. A value of *p* < 0.05 was considered statistically significant.

## 3 Results

### 3.1 Prion Infectivity in Muscle Tissue Derived From Prion‐Infected Mice

**Figure 1:**
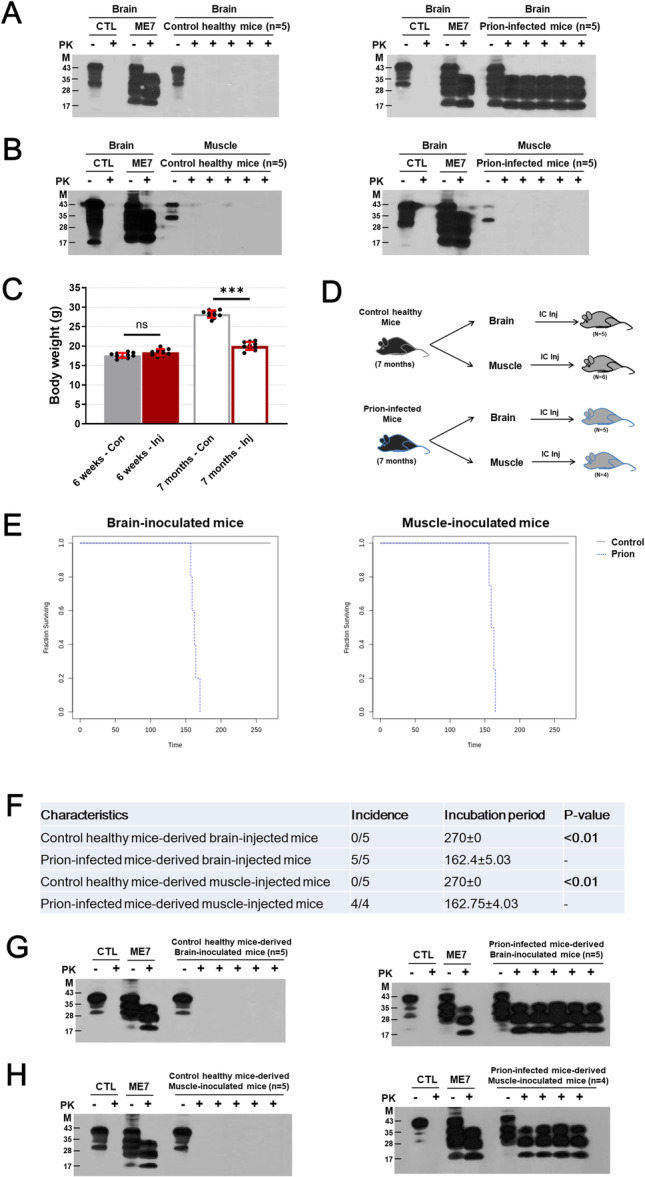
PrP^Sc^ evaluation of muscle tissue in prion‐infected mice. (A) PrP^Sc^ detection in brain‐inoculated mice. The left panel indicates western blot bands of PrP^Sc^ in homogenates of brain tissue from mice inoculated with homogenates of brain tissue from control healthy mice at 7 months postinjection (biological number, *n* = 5). The right panel indicates western blot bands of PrP^Sc^ in homogenates of brain tissue from mice inoculated with homogenates of brain tissue from prion‐infected mice at the end stage (biological number, *n* = 5). (B) PrP^Sc^ detection in muscle‐inoculated mice. The left panel indicates western blot bands of PrP^Sc^ in homogenates of muscle tissue from mice inoculated with homogenates of brain tissue from control healthy mice at 7 months postinjection (biological number, *n* = 5). The right panel indicates western blot bands of PrP^Sc^ in homogenates of muscle tissue from mice inoculated with homogenates of brain tissue from prion‐infected mice at the end stage (biological number, *n* = 5). (C) Measurement of body weights from control healthy and prion‐infected mice at 6 weeks and 7 months postinjection (biological number, *n* = 10). (D) Experimental design for the bioassay of prion infectivity using brain and muscle homogenates injected from control healthy and prion‐infected mice. “IC injection” indicates intracranial injection. (E) Survival analysis in brain‐ and muscle‐inoculated mice. The left panel indicates the survival rate versus days after intraperitoneal inoculation in mice inoculated with brain homogenates from control healthy and prion‐infected mice. The right panel indicates the survival rate versus days after intraperitoneal inoculation in mice inoculated with muscle homogenates from control healthy and prion‐infected mice. (F) Summary of Kaplan–Meier survival analysis results. Statistical significance was analyzed using the log‐rank test. (G) Bioassay results of brain‐inoculated mice. The left panel indicates western blot bands of PrP^Sc^ in homogenates of brain tissue from mice inoculated with homogenates of brain tissue from control healthy mice at 9 months postinjection (biological number, *n* = 5). The right panel indicates western blot bands of PrP^Sc^ in homogenates of brain tissue from mice inoculated with homogenates of brain tissue from prion‐infected mice at the end stage (biological number, *n* = 5). (H) Bioassay results of muscle‐inoculated mice. The left panel indicates western blot bands of PrP^Sc^ in homogenates of brain tissue from mice inoculated with homogenates of muscle tissue from control healthy mice at 9 months postinjection (biological number, *n* = 5). The right panel indicates western blot bands of PrP^Sc^ in homogenates of brain tissue from mice inoculated with homogenates of muscle tissue from prion‐infected mice at the end stage (biological number, *n* = 4). Data are presented as mean ± SD. Data were analyzed using Student's *t*‐test and one‐way ANOVA (***p < 0.001; ns, not significant, *p* > 0.05).

To determine the prion infectivity of muscle tissue in prion‐infected mice, we constructed a mouse model of prion disease using i.p. injection of the ME7 scrapie strain. As expected, PrP^Sc^ was detected in brain tissue from prion‐infected mice at 7 months postinjection, but not in the corresponding control healthy mice (Figure 1A). Although western blotting did not detect PrP^Sc^ bands in muscle tissue from prion‐infected mice (Figure 1B), these mice showed a significant reduction in body weight at 7 months postinjection (Figure 1C) and exhibited abnormal behaviors such as ataxia, muscle stiffness, and shivering (data not shown).

To further assess prion infectivity in muscle tissue, we performed a bioassay through i.c. injection of the muscle homogenates to detect amplified PrP^Sc^ (Figure 1D). Notably, all mice inoculated with homogenates of brain and muscle tissues from prion‐infected mice developed typical signs of prion disease, with incubation periods of 162.4 ± 5.03 and 162.75 ± 4.03, respectively. However, all mice inoculated with homogenates of the brain and muscle tissues from healthy control mice remained alive at 270 days postinoculation, at which point the experiment was terminated (Figure 1E,F). In addition, PrP^Sc^ bands were detected in brain tissue homogenates of mice inoculated with homogenates of the brain and muscle tissues from prion‐infected mice. However, no PrP^Sc^ bands were detected in homogenates of the brain tissue of mice inoculated with homogenates of brain and muscle tissues from corresponding control mice (Figure 1G,H). These findings indicated the presence of trace amounts of transmissible PrP^Sc^ in the muscle tissue from prion‐infected mice.

### 3.2 Prion Infection Causes Visceral Fat Depletion

**Figure 2:**
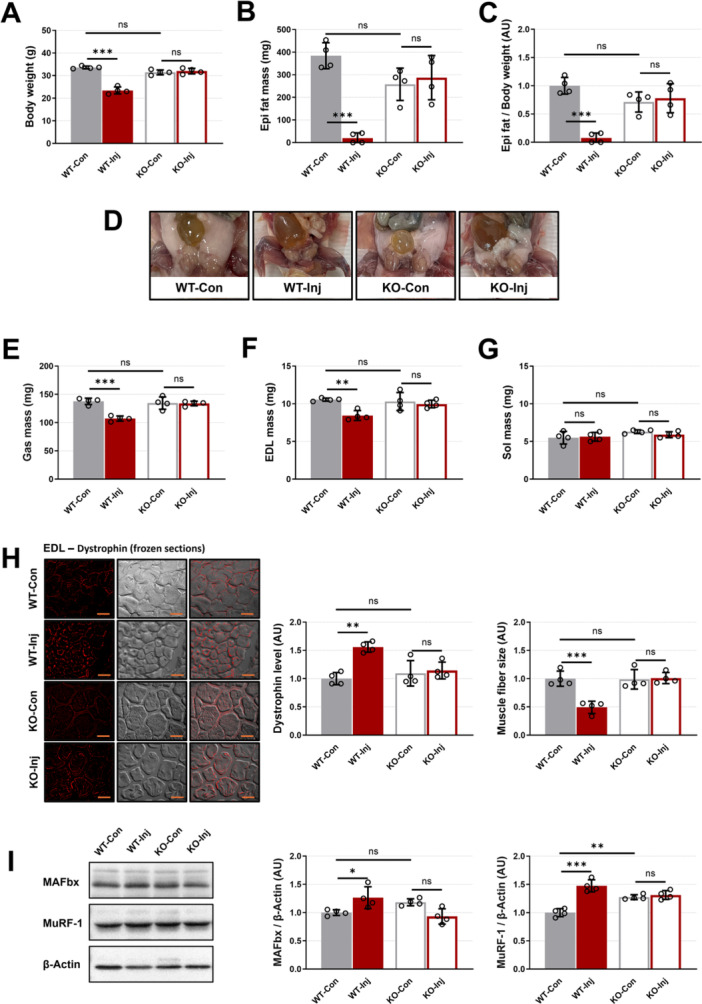
The effects of prion infection on body composition and skeletal muscle morphology at 7 months postinjection. (A) Body weight. (B) Epididymal (Epi) fat pad mass. (C) Epi fat pad mass to body weight ratio. (D) Representative images of visceral fat levels in mice. (E) Gastrocnemius (Gas) muscle mass. (F) Extensor digitorum longus (EDL) muscle mass. (G) Soleus (Sol) muscle mass. (H) Representative images of dystrophin immunofluorescence, dystrophin expression levels, and muscle fiber size in EDL (scale bar = 20 μm). (I) The effect of prion infection on the expression of MAFbx and MuRF‐1 in gastrocnemius muscle by analysis of western blot. Every group biological number, *n* = 4. Data are presented as mean ± SD. Data were analyzed using one‐way ANOVA (*p < 0.05; **p < 0.01; ***p < 0.001; ns, not significant, *p* > 0.05).

**Figure 3:**
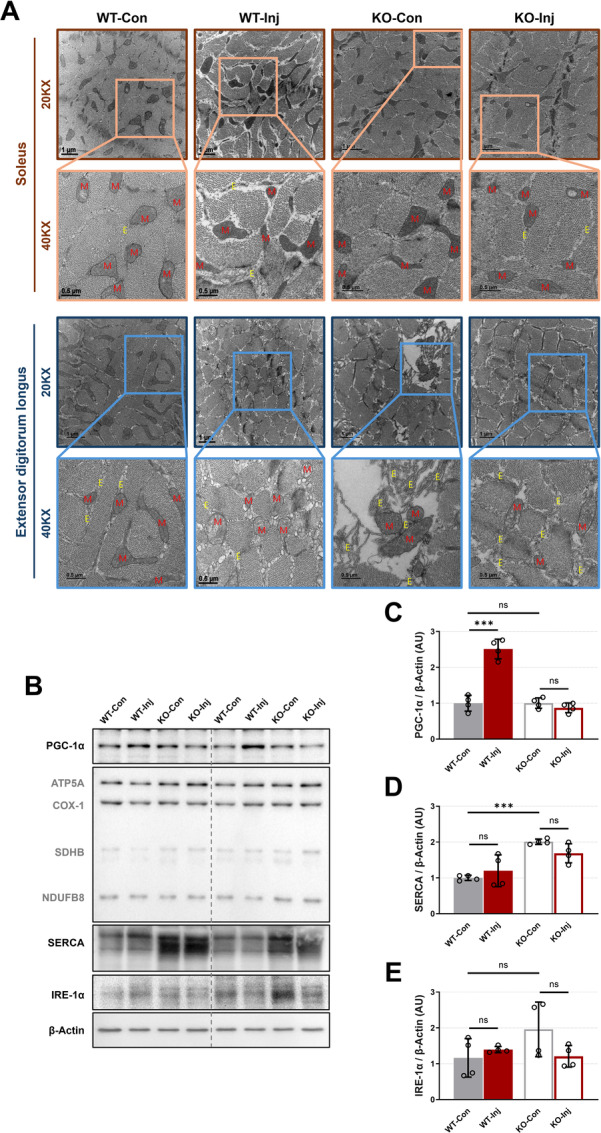
The effects of prion infection on skeletal muscle mitochondria and endoplasmic reticulum at 7 months postinjection. (A) Skeletal muscle mitochondrial and endoplasmic reticulum images were obtained in the EDL and soleus muscle using TEM analysis (20,000× image, scale bar = 1 μm; 40,000× image, scale bar = 0.5 μm); representative data are shown; E, endoplasmic reticulum; M, mitochondria. (B) Representative images of PGC‐1α, OXPHOS (NDUFB8, SDHB, COX‐1, and ATP5A), SERCA, IRE‐1α, and β‐Actin in gastrocnemius muscle by analysis of western blot. The expression levels of PGC‐1α (C), SERCA (D), and IRE‐1α (E) in gastrocnemius muscle by analysis of western blot. Every group biological number, *n* = 4. Data are presented as mean ± SD. Data were analyzed using one‐way ANOVA (***p < 0.001; ns, not significant, *p* > 0.05).

**Figure 4:**
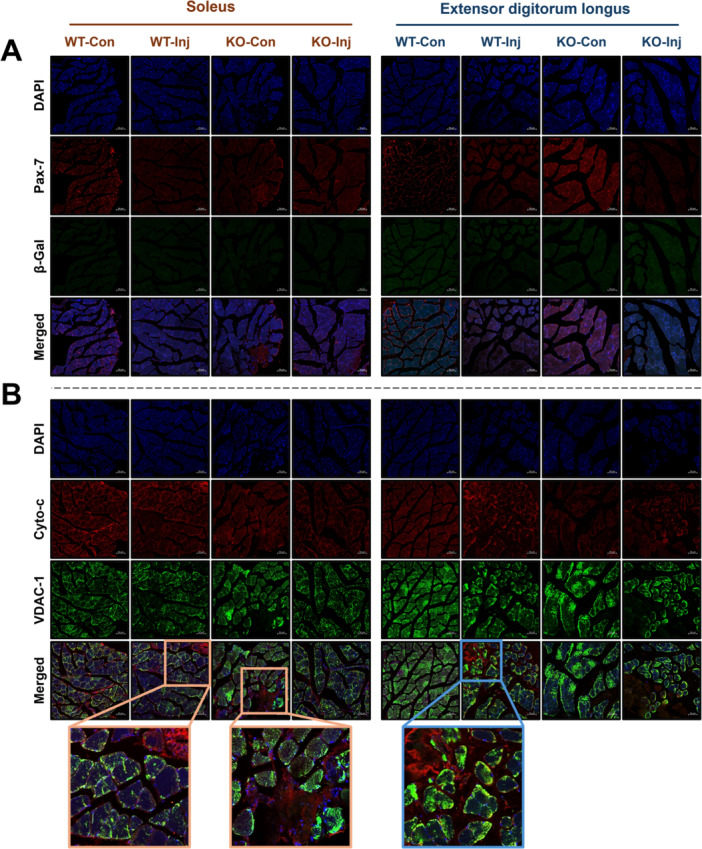
The effects of prion infection on skeletal muscle apoptosis and satellite cell senescence at 7 months postinjection. (A) The expression levels of Pax‐7/β‐galactosidase were measured in the EDL and soleus muscle by immunofluorescence; representative data are shown (scale bar = 50 μm). (B) The expression levels of Cyto‐c/VDAC‐1 were measured in the EDL and soleus muscle by immunofluorescence; representative data are shown (scale bar = 50 μm). Every group biological number, *n* = 3.

At 7 months after injection of the ME7 scrapie strain, we analyzed body composition and skeletal muscle samples of the wild‐type control (WT‐Con) group, wild‐type injection (WT‐Inj) group, knockout control (KO‐Con) group, and knockout injection (KO‐Inj) group. The results are shown in Figures 2, 3, 4.

Prior to sample collection, mice in the WT‐Inj group exhibited abnormal behaviors characterized by impaired motor coordination and tumor‐like symptoms. Furthermore, body weight measurements showed that wild‐type mice exhibited significant weight loss 7 months after injection, while no weight change was observed in the KO‐Inj group (Figure 2A). The measurements for visceral fat levels (Figure 2B,D) and fat percentage (Figure 2C) were consistent with the body weight results, with fat depletion observed only in the WT‐Inj group.

### 3.3 Muscle Fiber Specificity in Sarcopenia Caused by Prion Infection

Previous studies have shown that PrP^C^ protein expression in skeletal muscle is fiber‐type specific [9]. In order to more clearly illustrate the effects of prion infection on skeletal muscle, we collected samples from three representative sites: the Gas muscle (Type I, 10%–20%; Type IIa, 20%–30%; Type IIb/x, 50%–70%; Figure 2E), the EDL (Type IIb/x, 90%–95%; Figure 2F), and the Sol (Type I, 70%–80%; Type IIa, 20%–30%; Figure 2G).

The results of tissue mass analysis showed that the mass of Gas and EDL was significantly reduced in the WT‐Inj group (Figure 2E,F), while the weight of Sol was not affected by the infection of the prion (Figure 2G). We then further examined muscle fiber size and atrophy‐related factors in EDL and Gas that exhibited muscle atrophy. The frozen section staining results of EDL showed that the muscle fiber size in the WT‐Inj group decreased significantly, while the expression level of dystrophin protein (which has anti‐muscle atrophy function [10]) increased significantly (Figure 2H). This indicates that muscle atrophy induced by prion is not a genetic disorder, and that dystrophin protein expression levels increase protectively as muscle atrophy progresses. The expression levels of E3 ubiquitin ligase MAFbx and MuRF‐1, which are associated with protein degradation [11], were significantly increased in the WT‐Inj group (Figure 2I). This also indicates that prion‐induced muscle atrophy is mediated through enhanced ubiquitination pathways.

### 3.4 Prion Infection Induces Muscle Fiber Type‐Specific Mitochondrial Damage

The health of skeletal muscle mitochondria and ER plays a decisive role in maintaining the homeostasis of the skeletal muscle system [12]. To further elucidate the effects of prion infection on the skeletal muscle system, we performed transmission electron microscopy (TEM) analysis of Sol and EDL to examine the health status of organelles (Figure 3A).

TEM results showed that no significant large‐area mitochondrial damage (such as cristae loss and outer membrane damage [8]) or ER changes were observed in the Sol, but the WT‐Inj group showed large‐area mitochondrial damage in the EDL, and the KO group in the EDL showed ER stress (ER expansion in TEM results [13]) and varying degrees of mitochondrial morphological damage regardless of prion protein injection (Figure 3A).

Results from Gas showed that the expression levels of mitochondrial enzymes did not decrease despite morphological damage in all groups (Figure 3B). Conversely, the expression levels of PGC‐1α (a key regulator of mitochondrial biogenesis) significantly increased in the WT‐Inj group with more severe mitochondrial damage (Figure 3B,C). All KO groups showed significantly increased expression levels of SERCA (Figure 3B,D), an ER calcium signal‐related protein [8], consistent with the increased ER observed in EDL TEM images (Figure 3B).

### 3.5 *Prnp* Knockout Reduces Muscle Fiber Apoptosis and Atrophy Caused by Prion Infection

To evaluate the senescence status of satellite cells and apoptosis levels in tissues in each group, we performed immunofluorescence staining on Sol and EDL samples (Figure 4). The results obtained from confocal microscopy images showed that there were no significant differences in the expression levels of β‐galactosidase (β‐Gal; a marker of stem cell senescence [8]) among the groups. However, the expression level of Pax‐7 in the EDL of the KO‐Con group was significantly increased (Figure 4A). Apoptotic reactions were significantly increased in Sol and EDL samples from the WT‐Inj group, but no additional apoptotic changes were observed in the KO‐Inj group (Figure 4B).

## 4 Discussion

This study investigates the role of PrP^C^ in prion‐induced peripheral pathology, focusing on skeletal muscle. By using *Prnp* gene knockout mice, the authors examined how the absence of PrP^C^ affects the development of sarcopenia‐related symptoms following prion infection. Key pathological indicators, including body weight loss, fat depletion, muscle atrophy, mitochondrial damage, and cell death, were analyzed to determine the extent of peripheral toxicity. Moreover, PrP^Sc^ derived from muscle tissue may also participate in systemic or neuronal reinfection pathways.

Prion infection caused systemic metabolic disturbances in WT‐Inj mice, including significant body weight and visceral fat reduction, which were absent in KO‐Inj mice. This suggests that PrP^C^ mediates prion‐induced metabolic dysfunction. Skeletal muscle analysis revealed that type II fiber‐rich muscles (Gas and EDL) were more affected than the type I fiber‐rich soleus, likely due to differential PrP^C^ expression [9].

Histologically, WT‐Inj mice exhibited reduced muscle fiber cross‐sectional area and increased dystrophin expression, indicating nondystrophic muscle atrophy. Elevated levels of E3 ubiquitin ligases (MAFbx and MuRF‐1) implicate proteasome‐mediated degradation in muscle loss [14]. TEM further showed severe mitochondrial and ER damage in type II fibers of WT‐Inj mice, suggesting that PrP^Sc^ disrupts organelle function. *Prnp*‐knockout mice exhibited partial mitochondrial abnormalities and ER stress regardless of infection, indicating a physiological role for PrP^C^ in maintaining organelle homeostasis [15].

Interestingly, mitochondrial enzyme levels remained stable, while PGC‐1α expression increased in WT‐Inj mice, suggesting activation of compensatory mitochondrial biogenesis. In KO mice, SERCA expression was elevated, potentially maintaining ER calcium homeostasis in the absence of PrP^C^ [16, 17].

At the cellular level, *Prnp* deletion was protective. KO mice displayed elevated Pax‐7 expression, indicating preserved satellite cell function and delayed senescence [18]. TUNEL staining showed extensive apoptosis in WT‐Inj but not in KO‐Inj mice, highlighting PrP^C^'s role in prion‐induced cell death [19]. Meanwhile, even in uninfected states, *Prnp* knockout mice exhibit mild mitochondrial and ER abnormalities. This finding aligns with the observations of Liu and colleagues, reflecting the impact of physiological PrP^C^ loss on skeletal muscle function [8]. It should be noted that the protective phenotype observed in *Prnp* knockout mice may not exclusively result from the absence of PrP^C^‐dependent prion toxicity. Chronic PrP^C^ deficiency may also induce compensatory adaptations in mitochondrial and ER homeostasis, which could partially contribute to the resistance to prion‐induced muscle pathology observed in this model.

In conclusion, this study demonstrates that PrP^C^ is essential for mediating prion‐induced peripheral muscle pathology, particularly in type II fibers. Its deletion mitigates mitochondrial damage, proteolytic activation, satellite cell impairment, and apoptosis, offering a potential therapeutic target for prion‐related neuromuscular degeneration.

This study has several limitations. First, the sample size may have limited statistical power. Second, due to the lack of functional outcome data, the findings in this paper are more descriptively limited. Future research should provide mechanistic and functional validation of the findings presented in this paper.

## Author Contributions

Conceptualization: Byung‐Hoon Jeong, Sung Ho Kook, Sang Hyun Kim. Methodology: Byung‐Hoon Jeong, Sang Hyun Kim. Formal analysis: Wenduo Liu, Yong‐Chan Kim, Sae‐Young Won, Thi Thu Trang Kieu. Resources: Byung‐Hoon Jeong, Sung Ho Kook, Sang Hyun Kim. Writing – original draft: Wenduo Liu, Yong‐Chan Kim. Writing – review and editing: Byung‐Hoon Jeong, Sung Ho Kook, Sang Hyun Kim. Funding acquisition: Yong‐Chan Kim, Byung‐Hoon Jeong. All the authors have read and agreed to the published version of the manuscript.

## Ethics Statement

All experimental procedures were approved by the Institute of Animal Care and Use Committee of Jeonbuk National University (JBNU 2020‐080).

## Conflicts of Interest

The authors declare no conflicts of interest.

## Supporting information

Supporting File

## Data Availability

The data sets generated and analyzed during the current study, including original Western blot source files and transmission electron microscopy images, are available from the corresponding author upon reasonable request.
