# Supplementary material for: Prnp Deletion Mitigates Muscle Fiber Type‐Specific Sarcopenia Induced by Prion Infection in Mice
Source: Immun Inflamm Dis. 2026 Apr 13;14(4):e70425. doi: 10.1002/iid3.70425 (PMC13076917; doi:10.1002/iid3.70425)
Supplement: Supplementary file 1 — Supporting File [file IID3-14-e70425-s001.docx]

***Prnp* deletion mitigates muscle fiber type-specific sarcopenia induced by prion infection in mice**

Wenduo Liu^1,2#^, Yong-Chan Kim^3#^, Sae-Young Won^3^, Thi Thu Trang Kieu^4^, Sung Ho Kook^4^, Byung-Hoon Jeong^4,5*^, Sang Hyun Kim^2*^

^1^College of Physical Education, Beihua University, Jilin 132013, China

^2^Department of Sports Science, College of Natural Science, Jeonbuk National University, Jeonju 54896, Republic of Korea

^3^School of Life Sciences and Biotechnology, Gyeongkuk National University, Andong 36729, Republic of Korea

^4^Department of Bioactive Material Sciences, Research Center of Bioactive Materials, Jeonbuk National University, Jeonju 54896, Republic of Korea

^5^Korea Zoonosis Research Institute, Jeonbuk National University, Iksan 54531, Republic of Korea

^*^Address correspondence to: Byung-Hoon Jeong, Korea Zoonosis Research Institute, Jeonbuk National University, Jeonbuk, 54531, Republic of Korea, Phone: +82-63-900-4040, Fax: +82-63-900-4012, E-mail: [bhjeong@jbnu.ac.kr](mailto:bhjeong@jbnu.ac.kr)

Sang Hyun Kim, Department of Sports Science, College of Natural Science, Jeonbuk National University, Jeonju 54896, Republic of Korea, Phone: +82-63-270-2853, Fax: +82-63-270-4234, E-mail: [sh5275@jbnu.ac.kr](mailto:sh5275@jbnu.ac.kr)

^#^ These authors contributed equally to this work.

**Supplementary Information**

**Materials and Methods**

***Evaluation of PrP^Sc^ via western blotting analysis***

Brain and muscle tissues were obtained and homogenized with 10% volumes of Radioimmunoprecipitation assay (RIPA) Lysis Buffer (Thermo Fisher Scientific, USA) containing a protease inhibitor cocktail (Roche, Germany) and were centrifuged at 14,000 rpm for 15 min (4°C). The supernatants were collected and the protein concentrations were calculated using bicinchoninic acid assay kits (Bio-Rad, USA). To visualize the PrP^Sc^ band, samples were treated with 40 µg/mL of proteinase K for 1 h at 37°C. The samples were then boiled at 95°C for 10 min and loaded in a 12% sodium dodecyl sulfate gel. The loaded proteins were transferred to a nitrocellulose membrane (Amersham, USA) at 90 V for 100 min. The membranes were blocked in TBST containing 5% skim milk (Santa Cruz Biotechnology, USA) and were incubated at 4°C for 8 h with a mouse monoclonal anti-PrP antibody (SAF84). The membrane was rinsed in TBST and then was incubated with horseradish peroxidase-conjugated anti-mouse immunoglobulin G antibody (IgG) (Sigma-Aldrich, USA) for 1 h. After one more wash in TBST, the target bands were visualized by Pierce ECL Western Blotting Substrate (Thermo Fisher Scientific).

***Kaplan–Meier survival analysis***

Kaplan–Meier survival analysis was conducted with the survival and survminer packages of the R program (https://www.r-project.org/). Statistical significance was assessed using the log-rank test.

***Sample collection and tissue weight analysis***

Mice were anesthetized by intraperitoneal injection of a solution consisting of Zoletil (Virbac Laboratories), Rompun (Bayer Korea), and normal saline mixed at a ratio of 2:1:2 (1 mL/kg body weight). Following anesthesia, body weights were measured and tissue samples were collected. Bilateral Epi, Sol, EDL and Gas muscles were extracted for tissue weight analysis using an electronic balance. After measurement, tissues were either fixed in formalin, embedded in Tissue-Tek OCT compound (Sakura Finetek, USA) or frozen at -80°C.

***Immunofluorescence and confocal microscopy analysis***

Frozen tissue sections were fixed in 2% paraformaldehyde for 10 min, blocked for 60 min with 2% bovine serum albumin, 2% goat serum and 0.1% Triton X-100 in 1× phosphate-buffered saline (PBS). Alternatively, rehydrated paraffin-fixed muscle sections were permeabilized, blocked, and incubated with primary antibodies: β-Galactosidase (Cell Signaling, #27198, USA); Pax-7 (SCBT, sc-81648, USA); VDAC-1 (Abcam, ab15895, USA); Cytochrome-c (SCBT, sc-13560, USA); Dystrophin (SCBT, sc-73592, USA). After incubation with secondary antibodies (Anti-Rabbit Alexa Fluor 488, ab150077; Goat Anti-Mouse Alexa Fluor 594, ab150116, USA), the samples were mounted with DAPI-containing mounting medium (ChemCruz, sc-24941, USA) and analyzed using a super-resolution laser confocal scanning microscope [8].

***Biological transmission electron microscopy analysis***

TEM was used to examine the mitochondrial and ER structure of the soleus and extensor digitorum longus muscle. The muscle tissues were fixed with 2.5% glutaraldehyde and 4% formaldehyde in 0.1M phosphate buffer (pH 7.4) for 2 h, followed by post-fixation with 1% osmium tetroxide for 2 h. After dehydration in a graded ethanol series, the muscles were embedded in Epon-812 resin. Thin sections (~80 nm) were cut using a NOVA ultramicrotome (LKB, Vienna, Austria) and mounted on 100-mesh grids. The sections were stained with uranyl acetate and lead citrate and examined under an electron microscope (H7650, 80 kV, Hitachi, Japan). The TEM analysis was performed using a JEM-2010 microscope (JEOL) at the Center for University-Wide Research Facilities (CURF) of Jeonbuk National University.

***Western blot analysis***

Gas muscle, epi fat, and liver extracts were prepared and western blotting was performed as previously described [8]. The antibodies used are as follows: β-actin (Invitrogen, MA1-140, USA), PGC-1α (GeneTex, GTX37356, USA), OXPHOS (Abcam, ab110413, USA), MAFbx (SCBT, sc-166806, USA), MuRF-1 (SCBT, sc-398608, USA), SERCA (SCBT, sc-271669, USA), IRE-1α (SCBT, sc-390960, USA), mouse anti-rabbit (SCBT, sc-2357, USA) and goat anti-mouse (SCBT, sc-2005, USA).
